# Wound Complications Following Olecranon Fracture Fixation: Implant and Soft Tissue Considerations

**DOI:** 10.7759/cureus.41730

**Published:** 2023-07-11

**Authors:** Farhan Ahmad, Andre D Sabet, Mark Cohen, Rachel Hein, Marc Richard, Xavier Simcock, Robert Wysocki

**Affiliations:** 1 Orthopedic Surgery, Rush University Medical Center, Illinois, USA; 2 Orthopedics, Duke University Medical Center, Durham, USA

**Keywords:** wound breakdown, fracture, elbow, olecranon, re-operation, trauma, outcomes, open reduction internal fixation

## Abstract

Background: The aim of this multicenter, retrospective, case-control series was to investigate patient- and treatment-specific factors associated with wound breakdown following olecranon fracture fixation.

Methods: We identified patients at our two participating academic centers who were operatively treated for olecranon fractures and those who subsequently underwent a re-operation secondary to postoperative wound breakdown. Demographic and historical information was collected, including BMI and Charlson comorbidity index (CCI) scores. The primary outcome measure was the standardized radiographic measurement of plate prominence and soft tissue thickness posterior to the plate tip.

Results: We identified 32 patients who underwent internal fixation and subsequent wound breakdown. This was compared to a cohort of 35 matched controls that did not have wound issues. Cases with wound breakdown were of higher energy, nine being open cases compared to two in the control group (p<0.05). No differences were identified in plate prominence, soft tissue thickness, and plate type.

Conclusions: Wound breakdown following olecranon fracture fixation is more commonly seen in high-energy open injuries. Plate prominence, soft tissue thickness, and patient-specific factors do not correlate with wound breakdown. Further investigation into the factors influencing plate placement and how they may contribute to wound complications is needed.

## Introduction

Approximately 10% of elbow fractures involve the olecranon process of the ulna [1]. Olecranon fractures fall into a bimodal distribution, presenting secondary to high-energy injuries in the young and low-energy falls in the elderly. Typically, operative treatment is required for displaced fractures. Open reduction and internal fixation (ORIF) with plates is indicated for comminuted fractures, Monteggia fractures, fracture-dislocations, and oblique fractures extending distal to the coronoid [2]. A tension band technique is another option for simple transverse fractures with no comminution [3].

Olecranon fractures continue to have a higher rate of reoperation than other upper extremity fractures [4]. Reasons for this have not been well studied, and multiple factors have been suggested, some of which include infection or symptomatic hardware. Of particular interest is the influence of hardware type and positioning, which we sought to investigate in our study [4].

The nature of soft tissues in the posterior elbow can lead to unique challenges when operatively treating olecranon fractures. Due to the superficial location of the olecranon, along with the typically thin nature of the subcutaneous tissue, the absence of muscle over any internal fixation, the tendency to directly bear weight on the area, and incision placement directly over the olecranon, skin defects and wound breakdown are not uncommon following routine ORIF [5]. Wound breakdown at the olecranon quickly exposes hardware and bone and often requires complex surgical reconstruction to achieve reliable soft tissue coverage [6]. Knowledge of patient- and treatment-specific factors, if any, associated with wound breakdown after olecranon fixation would be useful to avoid this serious complication and the often complex reconstructions required to remedy the problem.

To the authors’ understanding, no study has been conducted with the primary aim of identifying specific factors that correlate with wound breakdown after olecranon fixation. Of particular interest is any evidence of patient factors (e.g., BMI, cardiovascular disease, other medical comorbidities), injury features (e.g., open vs. closed injuries, time to fixation), and implant features (e.g., tension band vs. plate fixation, the type of plate, plate prominence, soft tissue thickness from the plate) that may contribute to wound breakdown.

We hypothesized that an increased distance between the olecranon tip and the plate (plate prominence) and that some manufacturers' plates-especially those with increased proximal girth-would predispose patients to wound breakdown. We also hypothesized that decreased soft tissue thickness between the plate and the skin would lead to wound breakdown. However, obesity requires its own special consideration since, despite leading to thicker soft tissue envelopes, it is often independently linked to higher wound complications. Lastly, we postulated that most cases of significant wound breakdown would be in conjunction with plate, rather than tension band, fixation. We report our findings in this multicenter, retrospective case-control series of olecranon fractures.

## Materials and methods

We designed this as a two-institution study. As a part of our institution’s research review process, the aims and methodology of the study were reviewed and approved by the Director of Clinical Research Facilitation. Following institutional review board (IRB) approval at both Rush University Medical Center (approval no. 20083103-IRB01) and Duke University Medical Center, patients were identified using a search of diagnosis codes for muscle or adipofascial flaps or soft tissue rearrangement procedures (Current Procedural Terminology (CPT®) 15736, 14301, 14020). The list was cross-referenced for a history of open treatment for olecranon, Monteggia, and ulnar shaft fractures (CPT 24685, 24635, 25545) to generate a master list, which served as the inclusion criteria. Exclusion criteria were any history of a repeat olecranon fracture in the same arm, age less than 18, and fixation without a plate. Images were reviewed on all potential study patients to assure they had proximal ulna or olecranon fractures that were treated with pre-contoured olecranon plates or tension band wiring. Controls were generated from patients who had olecranon process fractures but no subsequent flap procedures, and each was assigned a number for randomized selection by a number generator. All procedures were performed either by fellowship-trained hand or orthopedic trauma surgeons.

**Figure 1:**
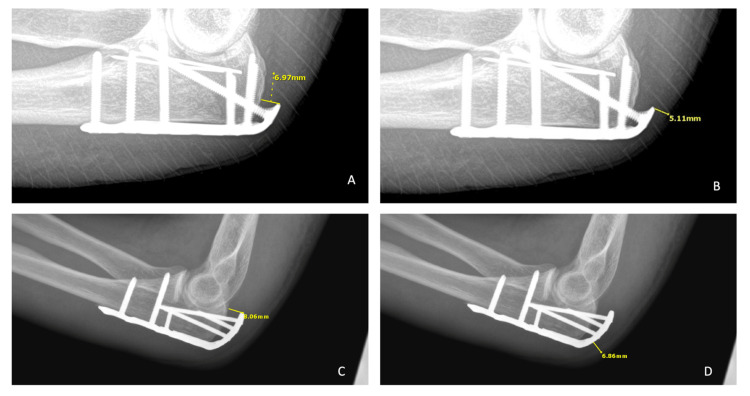
Sample of standardized radiographic measurements Synthes plate prominence (A) and soft tissue thickness (B); Acumed plate prominence (C) and soft tissue thickness (D)

Data collection included age, sex, BMI, Charlson comorbidity index (CCI) score, date of injury, date of surgery for fracture, time to initial surgery, open vs. closed injury, date of wound breakdown, date of surgery for soft tissue reconstruction, and type of reconstruction performed. Two fellowship-trained authors additionally independently reviewed final postoperative follow-up radiographs for each patient, recording the following data: type of implant used, measurement of plate prominence, and measurement of soft tissue thickness. Samples illustrating measurement methodologies are seen in Figure 1. Plate prominence measurements were defined as the length in millimeters of a line drawn parallel to the shaft portion of the plate from the posterior-most aspect of the proximal extension of the olecranon to the outermost metal of the plate, at the location along the proximal plate where that distance was the longest. Soft tissue thickness measurements were defined as the length in millimeters of a line drawn perpendicular to the plate itself from the outermost metal of the plate to the visible end of the soft tissue shadow superficially, at the location where that distance was the shortest. Measurements were made using the Opal-RAD picture archiving and communication system (Viztek LLC, Garner, NC, USA).

Surgical technique

Plate and screw fixation was performed by placing the plate on the dorsal (tension) side. Lag screws were additionally employed in cases of oblique fractures. Depending on the case, the plate was either placed as distally as possible, directly abutting the triceps or placed deep into the triceps via a longitudinal split in the tendon, which was laterally re-approximated as best as possible.

The tension band technique was performed by using either an 18-gauge wire or non-absorbable thick suture in a figure-of-eight manner through drill holes in the ulna in conjunction with two parallel 0.062-inch Kirschner wires, which were bent, cut short, and impacted deep into the triceps.

Statistical analyses

Upon completion of the database, statistical comparisons between cases and controls were performed. Categorical variables were reported by their frequency, while quantitative continuous variables were reported by the calculation of means and standard deviation. Unpaired Student’s t-test and Fisher’s exact testing were then used to compare means and frequencies between cases and controls with an alpha set to 0.05 [7]. Interobserver variability was also assessed by the calculation of paired Student’s t-test, means, and standard error of measurement.

Subgrouping by plate manufacturer Acumed (Hillsboro, OR, USA) vs. Synthes (West Chester, PA, USA) was then performed to examine correlations between the plate prominence and soft tissue thickness measurements. These manufacturers were chosen since all cases of olecranon plating were performed using one of these two plating systems.

## Results

There were 67 patients included in this study (42 females and 25 males) with a mean age of 49 ± 21 years, out of which 32 (17 females and 15 males) had wound breakdown. The control group comprised 35 patients (25 females and 10 males) with no wound problems after olecranon fracture fixation. Body mass index (BMI) averaged 25 ± 6 kg/m2. The CCI averaged 2 ± 2 points. The Student’s t-test for these demographic data was not significantly different between cases and controls (p>0.05) implying homogenous population sampling. A summary of demographic information by cases and controls is in Table 1.

**Table 1:** Demographic and historical information of cases and controls CCI: Charlson comorbidity index

	Cases (n=32)	Controls (n=35)	Total (n=67)
Average age ± 1 Standard deviation	50 years old ± 22	48 years old ± 20	49 years old ± 21
Sex	17 female, 15 male	25 female, 10 male	42 female, 25 male
Average BMI ± 1 Standard deviation	24 kg/m^2^ ± 7	25 kg/m^2^ ± 5	25 kg/m^2^ ± 6
Average CCI score ± 1 Standard deviation	2 ± 3	2 ± 2	2 ± 3

The following injury and surgery characteristics are summarized in Table 2. Initial injuries were closed in 56 patients and open in 11. The time to initial surgery averaged 7 ± 5 days. An additional procedure at the time of index olecranon fixation was performed in 16 patients (e.g., triceps repair, lateral collateral ligament repair, radial head arthroplasty, etc.). Fisher’s exact testing between cases and controls was statistically significant (p<0.05) for initial injury type (cases had 23 closed and 9 open, while controls had 33 closed and 2 open). Table 2 illustrates these statistically significant differences. Additional procedures aside from olecranon fixation were performed in 28% of the case group vs. 20% in the control group, but this was not statistically significant (p>0.05). The average nine-day span from injury to initial surgery in the cases did not statistically differ (p>0.05) from the six days in the controls.

**Table 2:** Injury and surgery characteristics of cases and controls

	Cases (n=32)	Controls (n=35)	Total (n=67)
Open injury	9	2	11
Closed injury	23	33	56
Average time to initial surgery ± 1 Standard deviation	9 days ± 6	6 days ± 3	7 days ± 5
Additional procedures at index	9	7	16

The distribution of plates by the company was: 34 Acumed, 27 Synthes. Tension bands were used in four patients, three of which were in conjunction with a plate and in the control cohort. There were no cases of soft tissue breakdown requiring reoperation in patients with isolated tension band constructs. In the 32 cases, the time from fracture fixation to reported wound breakdown was 37 ± 30 days (median 32 days). The time from reported wound breakdown to a soft tissue procedure was 27 ± 54 days (median 7.5 days).

The mean radiographic measurements are summarized in Table 3. Interobserver variability was low, as evidenced by a nonsignificant paired Student’s t-test (p>0.05) for interobserver differences in means and standard errors of measurement. The mean plate prominence was 6 ± 2 mm (5 ± 2 mm in cases, 7 ± 2 mm in controls). The mean soft tissue thickness was 5 ± 3 mm (5 ± 3 mm in cases, 5 ± 3 mm in controls).

**Table 3:** Radiographic measurements of plate prominence and soft tissue thickness

	Cases (n=32)	Controls (n=35)	Total (n=67)
Average plate prominence ± 1 Standard deviation	5 mm ± 2	7 mm ± 2	6 mm ± 2
Average soft tissue thickness ± 1 Standard deviation	5 mm ± 3	5 mm ± 3	5 mm ± 3

The results of subgroup analyses on radiographic measurements are summarized in Table 4. Subgrouping by additional procedure at index (yes vs. no) was not correlated with plate prominence or soft tissue thickness (p>0.05). Subgrouping by plate manufacturer (Acumed vs. Synthes) revealed statistically significant differences in plate prominence (p<0.05), with Synthes plates having greater plate prominence than Acumed plates. Soft tissue thickness measurements, however, did not show statistically significant differences between manufacturers (p>0.05).

**Table 4:** Summary of subgroup analyses and correlations with radiographic measurements

	Average plate prominence ± 1 Standard deviation	Average soft tissue thickness ± 1 Standard deviation
No additional procedure at index	6 mm ± 2	5 mm ± 3
			Additional procedure at index	7 mm ± 2	5 mm ± 2
						Synthes plate	5 mm ± 2	4 mm ± 2
Acumed plate	7 mm ± 2	6 mm ± 3			

## Discussion

This study found that plate type and prominence up to 9 mm did not correlate with cases of wound breakdown after olecranon fracture fixation. Our initial hypothesis was that the plate manufacturer, with regard to plate thickness and the patient’s soft tissue anatomy, would correlate with wound breakdown. Additionally, we theorized that patient-specific factors, such as medical comorbidities, would correlate with wound breakdown. Nonetheless, we did not find any patient-specific factors that contributed to wound breakdown, nor did we see any relationship in our radiographic assessments of plate prominence and soft tissue thickness between cases and controls.

By investigating wound breakdown specifically, this study presents a pertinent negative result regarding plate prominence using a large, multicenter case-control series. As defined by Horan et al., wound breakdown meets the definitional criteria for a deep incisional surgical site infection, and at the posterior elbow especially, wound breakdown is a serious complication that typically requires reoperation and often flap coverage [8]. Therefore, we sought to investigate if any factors contributed to this adverse outcome. Results from this study have the potential to inform, improve, and guide patient care, as no study to the authors’ knowledge has examined a cohort of patients who experienced wound breakdown following olecranon fracture fixation, nor to our knowledge has it identified plate position as a possible factor in developing wound breakdown.

Previous studies on olecranon fractures have generally investigated outcomes and complications for different fixation methods [9]. The literature appears to support a low complication rate for all fixation methods (ORIF with plating vs. tension band wiring), with mixed results on whether plating has a higher association with infection [10,11]. To the authors’ knowledge, there have only been two prospective randomized studies reporting infectious complications by fixation method for olecranon fractures. Duckworth et al. reported a 13% infection rate (n=4/32) with plates and 0% (n=0/30) with tension bands. Of the four patients with infection, two underwent reoperation [12]. No mention of wound breakdown was reported, nor was there any use of a flap procedure. In a prospective trial, Hume (&) Wiss found no difference in complication rate between 41 patients with either plates or tension bands [13]. They similarly reported no frank breakdown or need for a flap procedure. Results from these studies, and a select number of retrospective studies, are summarized in Table 5, all of which suggest a low infection rate regardless of the method of fixation.

**Table 5:** Reported infection rates comparing plates and tension bands

Author	Design	Journal	N	Infection rate	Flap procedure rate
Duckworth et al., 2017 [12]	Prospective	Journal of Bone & Joint Surgery	32 plates, 30 tension bands	13% (4) plates, 0% (0) tension bands	0% (0)
Hume (&) Wiss, 1992 [8]	Prospective	Clinical Orthopaedics & Related Research	3 plates, 37 tension bands, 1 screw	0% (0) plates, 3% (1) tension bands	0% (0)
Tarallo et al., 2014 [14]	Retrospective	Archives of Orthopaedic and Trauma Surgery	45 plates, 33 tension bands	0% (0) plates, 3% (1) tension bands	0% (0)
Amini et al., 2015 [15]	Retrospective	American Journal of Orthopaedics	10 plates 10 tension bands	0% (0) plates, 0% (0) tension bands	0% (0)
Schliemann et al., 2014 [16]	Retrospective	Acta Orthopaedica	13 plates 13 tension bands	8% (1) plates 8% (1) tension bands	0% (0)

It should be noted that we had no cases of wound breakdown requiring reoperation for soft tissue coverage in conjunction with tension band fixation. While we do not have a denominator of how many plates vs. tension band constructs were performed in the database from which these patients were drawn, tension band wiring may be somewhat protective against this complication. Reasons could include more simple fracture patterns amenable to the use of a tension band construct and less soft tissue injury, creating a favorable environment for wound healing. It also may be that, while the knots from tension bands can cause local pain, it is the broadly increased thickness of a plate that brings an increased risk of wound breakdown.

The mean time to wound breakdown in our study was 37 days and was reported as soft tissue breakdown with no documentation of surgical site infection. This is potentially reflective of the theory that soft tissue breakdown represents the equivalent of pressure sores over a posterior plate. It is also common for the soft tissue breakdown to occur not on the incision itself but adjacent to the incision and directly over the plate. For this reason, at our institutions, we take steps to limit posterior pressure on the elbow for at least the first six weeks postoperatively. This includes avoiding an orthosis that applies direct pressure to the olecranon. We favor the use of an anterior thermoplastic splint or a posterior splint with a window over the olecranon and strictly direct the patient and family on how to elevate the arm with the shoulder abducted so weight is resting on the medial rather than the posterior elbow. Patients are also routinely instructed to strictly avoid armrests, desks, or tabletops that would apply posterior pressure. Future studies should evaluate the role of patient perception and activity levels on the risk of developing wound breakdown during the postoperative period, as well as the ideal steps in the prevention of the complication via patient counseling and the choice of postoperative orthosis.

Additionally, we found that cases of wound breakdown had a greater frequency of open fractures, which was statistically significant (p<0.05). Open injuries typically represent high-energy trauma and involve greater soft tissue disturbances and rates of infection than closed injuries, which may contribute to the increased susceptibility for wound breakdown. Potential reasons could include a more significant soft tissue injury resulting in a poor wound healing environment and a compromised blood supply to this area. Future studies should include incisional placement with consideration of the blood supply to the soft tissue envelope of the posterior elbow.

Regarding patient-specific factors affecting outcomes after olecranon fracture fixation, while numerous studies have assessed patient factors and correlated them with various patient-reported outcomes, none have drawn any conclusions on associations with soft tissue healing. Our study did not find any patient-specific factors or co-morbidities that correlated with wound breakdown after ORIF.

This study was limited by its retrospective design. While we had a large sample size in comparison to the existing literature, our results may still have been limited in statistical power given the low rate of wound breakdown experienced after ORIF of olecranon fractures. Large database studies, perhaps using national registries, could provide more clarity. Additionally, while our control group was randomly selected and taken from the full cohort of olecranon fractures treated, this process may have introduced a form of selection bias. However, this matching process was key to reducing confounding by other variables.

## Conclusions

Plate prominence is not a predominant factor in wound breakdown. However, wound breakdown is more commonly seen in higher-energy open injuries. Patient-specific factors, such as BMI and medical comorbidity burden, also do not correlate with breakdown. Future studies should assess the role of plate placement and soft tissue compromise prior to fixation, as well as patient perceptions and activity levels during the postoperative period.
